# Lactate Levels and Clearance: Key Predictors of Prognosis for COVID-19 and Non-COVID-19 Septic Shock Patients in the Emergency Department

**DOI:** 10.3390/clinpract14030065

**Published:** 2024-05-09

**Authors:** Cosmin Iosif Trebuian, Octavia Maria Brici, Dumitru Sutoi, Daian Ionel Popa, Daniel Raul Chioibas, Ovidiu Alexandru Mederle

**Affiliations:** 1 Department of Surgery I, “Victor Babes” University of Medicine and Pharmacy, E. Murgu Square No. 2, 300041 Timisoara, Romania; trebuian.cosmin@umft.ro (C.I.T.); dumitru.sutoi@umft.ro (D.S.); chioibas.raul@umft.ro (D.R.C.); mederle.ovidiu@umft.ro (O.A.M.); 2 Department of Doctoral Studies, “Victor Babes” University of Medicine and Pharmacy, E. Murgu Square No. 2, 300041 Timisoara, Romania; daian-ionel.popa@umft.ro

**Keywords:** septic shock, lactate clearance, critical care, emergency department

## Abstract

Background: This investigation assesses the prognostic value of lactate levels and their clearance in septic shock patients, particularly emphasizing the comparative analysis between COVID-19 and non-COVID-19 patients in the emergency department. This study aims to elucidate the unique prognostic implications of lactate dynamics in these distinct patient groups, thereby enhancing the management of septic shock. Methods: An observational prospective study was conducted, enrolling 114 septic shock patients from the Emergency County Hospital Resita, Romania, categorizing them into COVID-19 and non-COVID-19 groups to examine their initial lactate levels, clearance rates, and their correlation with patient outcomes. Results: This study identified significant differences in the initial lactate levels and clearance rates between the two groups, indicating higher initial lactate levels and slower clearance rates in COVID-19 patients. Survivors demonstrated significantly lower initial lactate levels (1.5 ± 0.4 mmol/L) and higher lactate clearance rates (33 ± 15%) compared to non-survivors (2.5 ± 0.5 mmol/L and 24 ± 9%, respectively; lactate levels *p* = 0.001, clearance rates *p* = 0.002). Conclusions: Lactate monitoring, particularly clearance rates, is crucial in the prognostic assessment of septic shock patients. These findings highlight the need for targeted interventions in COVID-19 patients to improve outcomes, underscoring lactate dynamics as a vital component of septic shock management in differing patient populations.

## 1. Introduction

Sepsis is a life-threatening syndrome that leads to hemodynamic instability, and is defined as a life-threatening organ dysfunction secondary to the host’s dysregulated response to infection [1,2]. Considering its high incidence in the population and its high impact on hospital mortality, sepsis represents a significant threat. Additionally, sepsis management brings significant costs [3]. Sepsis exhibits a time-dependent pathology with a high mortality, reaching around 10% in the case of sepsis, and rising above 40% in patients with septic shock [4].

It is estimated that there are 189 cases of sepsis treated in hospitals per 100,000 per-son years, with a mortality rate of 26.7%. The estimated incidence of sepsis requiring admission to the medical and surgical intensive care units (ICU) was 30%, of which 41.9% died before hospital discharge [5].

Acute medical care in hospital emergency departments has experienced rapid development in recent years and has gained increasing importance not only from a professional medical point of view, but also from an economic and health policy perspective [6]. Emergency departments have always been the first point of contact for hospitals in many situations, including man-made and natural disasters. The coronavirus disease 2019 (COVID-19) pandemic has brought a changing landscape and new challenges in emergency departments too, these being the first places where patients with symptoms of COVID-19 were met in health institutions. Emergency departments play an important role in diagnosing the disease and isolating and hospitalizing patients if necessary [7]. During the COVID-19 pandemic, a large number of critically ill and severe COVID-19 patients met the diagnostic criteria for sepsis and even septic shock [8,9]. From the clinical point of view, patients with this type of shock may present high cardiac output, hypotension, a large pulse pressure, a low diastolic pressure, and warm extremities with good capillary refill. These findings on physical examination strongly suggest a working diagnosis of septic shock. Also, routine laboratory tests are performed for all inpatients [10] and outpatients. These tests are useful for both diagnosis and diseases progression monitoring [11]. The following blood tests are routinely used in the evaluation of septic shock: complete blood count (CBC), C-reactive protein (CRP), clinical chemistry, and arterial blood gas [12]. Host-response and pathogen-specific biomarkers have been described in the literature as having utility in staging disease severity, prognosis, and the response to treatment. In addition to CRP, some common host-response biomarkers used in regular practice include procalcitonin (PCT) and lactate [13]. However, many other biomarkers have been discovered to play a role in sepsis, including complement, cytokines, chemokines, damage-associated molecular patterns (DAMPs), calprotectin, and E-selectin. Their use in daily practice is limited, and more research is needed to identify the combinations of biomarkers that can impact diagnosis and treatment and improve patient outcomes [14].

Regardless of its etiology, lactate’s presence and trends over time have been studied in sepsis and other disease states and has been shown to be independently associated with increased mortality [15,16]. There are literature data which claim that the prognostic accuracy of serial lactate levels suggests that lactate clearance may be a useful therapeutic target for resuscitation [17]. Additionally, recent findings reveal that lactate clearance is associated with an improved outcome across several cohorts of critically ill patients. Lactate levels and central venous oxygen saturations are frequently discordant. The treatments for COVID-19 patients with sepsis were very limited. Accordingly, the therapeutic management of these patients was conducted considering that for sepsis, improving ventilation is one of the key points [8]. Targeting lactate clearance as part of a quantitative resuscitation strategy may be as effective as targeting central venous oxygen saturation [18].

In addressing the critical challenge of managing septic shock in the emergency department, our study focuses on the pivotal role of lactate levels and clearance as prognostic markers. Given the heightened complexities introduced by the COVID-19 pandemic, distinguishing between affected and unaffected patients becomes crucial. We aim to dissect the prognostic value of lactate dynamics in septic shock, with a comparative lens on COVID-19 versus non-COVID-19 patients. This study addresses a critical gap in the current understanding and the management of septic shock, providing targeted insights that could potentially improve emergency care practices. This clarification aligns with our investigation of contemporary research needs, as highlighted by recent studies such as those conducted by Carpenè et al. [19] and Bruno R. [20].

## 2. Materials and Methods

*Study Design:* This observational prospective study was conducted in the Emergency Department (ED) of the Emergency County Hospital Resita, Romania. The study period spanned from 1 October 2021 to 31 September 2022. During these periods, we aimed to evaluate the prognostic significance of the lactate levels and clearance in patients presenting with septic shock. Septic shock was defined according to the Third International Consensus Definitions for Sepsis and Septic Shock (Sepsis-3) [4], which includes the presence of sepsis with persistent hypotension requiring vasopressors to maintain a mean arterial pressure (MAP) of 65 mmHg or greater and having a serum lactate level greater than 2 mmol/L, despite adequate volume resuscitation.

**Figure 1:**
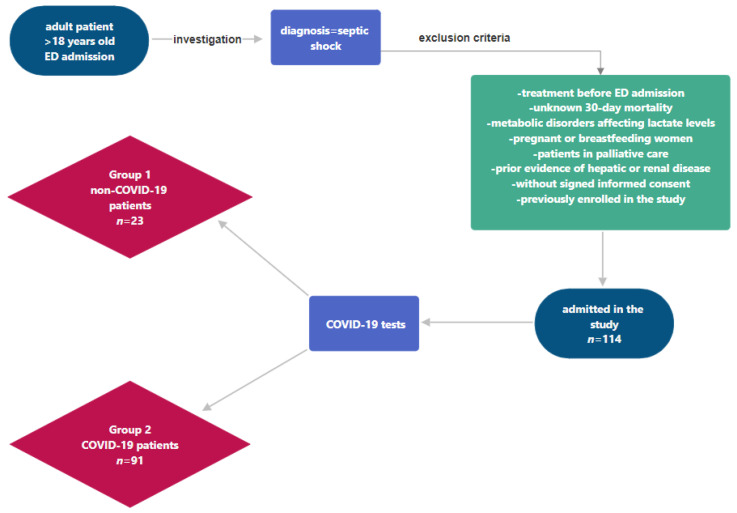
Patient inclusion criteria.

*Patients*: The inclusion criteria were patients older than 18 years who were diagnosed with septic shock at the time of presentation to the ED (Figure 1). Patients were randomly selected and included in the study after a primary examination. We excluded from this study patients who had received significant intravenous fluid or vasopressor treatment before ED admission presentation, as such interventions could alter baseline lactate levels, also, patients with an unknown 30-day mortality were excluded. Additionally, individuals with metabolic disorders known to independently affect lactate levels, such as mitochondrial disorders or liver diseases, were omitted to avoid confounding results. Pregnant or breastfeeding women were excluded due to the physiological changes affecting lactate metabolism and fluid balance during these conditions. Patients in palliative care, where aggressive treatment strategies for septic shock were not pursued, were also excluded. Patients with known hepatic or renal impairment were evaluated separately to determine the impact of these conditions on lactate clearance and were excluded from this study. Furthermore, this study did not include patients who were unable to understand the study procedures or provide informed consent when no translation services were available, as well as individuals previously enrolled in the study to prevent duplicate data collection. A total of 114 patients who presented at the ED were successfully enrolled in this study. All the patients admitted in the ED during the defined time period were tested for COVID-19 infection (all patients were tested using the PCR—Polymerase Chain Reaction—method using nasopharyngeal swabs). According to their COVID-19 infection status, these patients were subsequently divided into two groups: Group 1—non-COVID-19 patients—and Group 2—COVID-19 patients.

*Intervention and Monitoring:* Upon admission, all patients underwent volemic resuscitation within the first hour, receiving 10–20 mL/kg of crystalloid solutions based on their fluid status. Arterial blood gases (ABGs) were monitored, with a particular focus on parameters that correlate with lactate levels, such as pH and base excess (BE). To maintain a mean arterial pressure (MAP) over 65 mmHg, a combination of Noradrenaline and Dobutamine was administered as needed. The median time from admission to intensive care unit (ICU) transfer was recorded. The prognostic power of lactate levels was evaluated at multiple key points: upon admission, at 24 and 48 h post-admission, and at discharge or death. Lactate clearance was calculated based on the difference in lactate levels measured upon ED admission and at 24 h post-admission. This interval was chosen to provide a strong indicator of the patient’s initial response to treatment and metabolic recovery. These intervals were chosen to assess both the immediate and short-term outcomes related to lactate clearance in the context of septic shock management.

*Data Collection:* Data were collected on patient demographics, clinical presentations, ABG parameters, lactate levels at admission, and subsequent intervals to assess lactate clearance. The lactate clearance was calculated based on the difference between initial lactate levels upon ED presentation and follow-up measurements. Lactate levels were initially measured upon admission and subsequently at 6, 24, and 48 h. Lactate clearance was calculated as the percentage reduction from the initial value, which is considered to be a robust indicator of improvement in the patient’s metabolic status and response to treatment. As well, a 30 day-designated timeframe for an in-hospital mortality assessment was established.

*Statistical Analysis:* Statistical analysis was performed using MedCalc^®^ Statistical Software version 20.118 (MedCalc Software Ltd., Ostend, Belgium; 2022). The primary outcome measure was the association between the lactate levels (and clearance rates) with patient outcomes, including the ICU length of stay, ventilator-free days, and mortality. Descriptive statistics were used to summarize patient characteristics. Comparative analyses were conducted using the Chi-square test for categorical variables and the Student’s *t*-test or the Mann–Whitney U test for continuous variables, as appropriate. Logistic regression analyses were utilized to explore the impact of variables like lactate levels on binary outcomes such as survival. Additionally, Kaplan–Meier survival analysis was applied to examine the survival probabilities over the study period, differentiating between COVID-19 and non-COVID-19 patients. A *p*-value of <0.05 was considered statistically significant.

## 3. Results

Our study included a total of 114 patients aged between 23–91 years who presented with septic shock at the Emergency Department, 23 patients being included in the non-COVID-19 group (Group 1) and 91 patients being included in the COVID-19 group (Group 2). The average age of the non-COVID-19 patients was 67 years (SD = 18.41), which was slightly lower than the COVID-19 group, which had an average age of 71 years (SD = 15.85), (*p* = 0.031). The gender distribution was similar between the two groups, with 74% of the non-COVID-19 group and 73% of the COVID-19 group being male, (*p* = 0.712).

In analyzing comorbidities and complications among patients with septic shock, our study highlighted several key findings based on COVID-19 status (Table 1). Among the notable differences, heart failure was marginally more prevalent in COVID-19 patients (9%) compared to non-COVID-19 patients (4%), (*p* = 0.045). Cardiovascular diseases (CVDs) showed a marked contrast, which was significantly more frequent in non-COVID-19 patients (91%) than in those with COVID-19 (29%), with a notable *p*-value of 0.021. Obesity also presented a significant difference, being more common in non-COVID-19 patients (30%) versus COVID-19 patients (18%), (*p* = 0.031). Furthermore, the incidence of multi-organ failure was significantly higher in the COVID-19 group (10%) compared to the non-COVID-19 group (4%), (*p* = 0.001). Other conditions, such as COPD, CKD, diabetes, hematologic, and neurological diseases, although varied in prevalence, did not show statistically significant differences between the groups.

**Figure 2:**
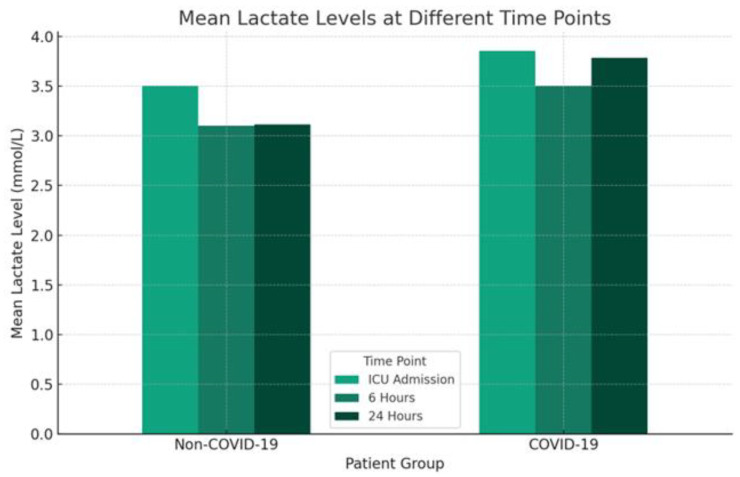
Mean lactate levels at different time points for septic shock patients. This bar graph displays the average lactate levels measured at ICU admission 6 h, and 24 h post-admission for septic shock patients, categorized by COVID-19 status. The *x*-axis indicates the Non-COVID-19 (**left** bars) and COVID-19 (**right** bars) patient groups and the *y*-axis shows the mean lactate level in mmol/L. Each time point is represented by a different color within the group bars, illustrating distinct lactate dynamics across the two patient groups.

In analyzing the resulted laboratory parameters during the first 48 h after admission, significant differences were noted in the lactate levels at various time points, highlighting the impact of COVID-19 on metabolic stress response. Initially, COVID-19 patients presented with higher lactate levels (6.2 ± 2.9 mmol/L) compared to non-COVID-19 patients (4.7 ± 2.6 mmol/L), (*p* = 0.001) (Table 2). This pattern persisted across subsequent measurements at 6, 24, and 48 h, with *p*-values of 0.021, 0.037, and 0.046, respectively, indicating consistently higher lactate levels in the COVID-19 group. The differences in lactate dynamics in both groups are illustrated in Figure 2.

Lactate clearance rates, an indicator of recovery from shock, showed a marginal difference at 6 h (*p* = 0.054) but reached statistical significance at 24 h, with non-COVID-19 patients demonstrating a slightly better clearance (*p* = 0.017). Although white blood cell count (WBC) and C-reactive protein (CRP) levels were elevated in both groups, differences did not reach statistical significance (*p* = 0.078 and *p* = 0.064, respectively). However, procalcitonin (PCT) levels were significantly higher in COVID-19 patients (17.2 ± 3.7 ng/mL vs. 9.7 ± 4.1 ng/mL, *p* = 0.049), suggesting a more pronounced inflammatory response (Table 2).

The study also observed a minor, yet significant, difference in the partial pressure of oxygen (pO_2_) levels (*p* = 0.045), with non-COVID-19 patients exhibiting slightly higher levels, potentially indicating a less severe respiratory compromise in this group. Other parameters such as pH and the partial pressure of carbon dioxide (pCO_2_) showed no significant difference, underscoring the complexity of the metabolic and respiratory interactions in septic shock across different patient populations.

A significant proportion of patients required respiratory support, with approximately 67% of patients needing high-flow oxygen therapy, 56% of patients requiring CPAP (Continuous Positive Airway Pressure), and 59% of patients undergoing orotracheal intubation. The need for such advanced respiratory support was indicative of the severe respiratory compromise associated with septic shock, further complicated by COVID-19 in some cases. The median ICU hospitalization period, as inferred from the data, was approximately 3.7 days, indicating a rapid progression to either recovery or deterioration among this patient population.

Our findings illustrate a marked association between lactate dynamics and survival outcomes in septic shock patients. The overall 30-day mortality rate was 37% for the entire cohort of 114 patients, both COVID-19 and non-COVID-19, particularly pronounced among those with COVID-19. For Group 1—the non-COVID-19 patients—the mortality rate was 25%. In contrast, Group 2—the COVID-19 patients—experienced a higher mortality rate of 42%. Table 3 delineates the differences in lactate levels and clearance rates between survivors and non-survivors 72 h post-admission. Survivors demonstrated significantly lower initial lactate levels (1.5 ± 0.4 mmol/L) and higher lactate clearance rates (33 ± 15%) compared to non-survivors (2.5 ± 0.5 mmol/L and 24 ± 9%, respectively; lactate levels *p* = 0.001, clearance rates *p* = 0.002). These observations underscore that both lower initial lactate levels and effective lactate clearance are predictive of better outcomes in septic shock patients, emphasizing the critical role of early lactate monitoring.

The initial lactate levels were found to be a significant predictor of mortality, with higher levels associated with higher odds of 30-day mortality (OR = 0.60, 95% CI-0.55 to 0.66, *p* < 0.001). Similarly, 24 h lactate clearance was significantly associated with survival, with each 10% increase in lactate clearance decreasing the odds of 30-day mortality by 20% (OR = 0.44, 95% CI-0.41–0.47, *p* = 0.002).

**Figure 3:**
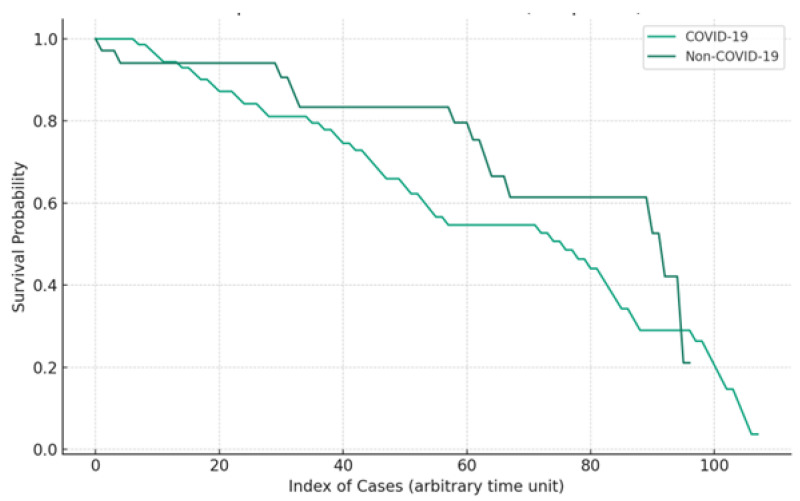
Kaplan–Meier survival curves for septic shock patients. This figure illustrates the survival probabilities over time for patients with septic shock, differentiated by COVID-19 status. The *x*-axis represents the index of cases, serving as an arbitrary time unit, and the *y*-axis indicates the survival probability.

Kaplan–Meier survival curves (Figure 3) further support the prognostic value of early lactate dynamics. The analysis delineates survival probabilities over time, differentiating between COVID-19 and non-COVID-19 patients. This graphically illustrates that effective lactate clearance within the first 24 h significantly correlates with improved survival outcomes.

The Kaplan–Meier survival curves represented in Figure 2 show the progression of patient survival over the 30-day study period. The ‘index of cases’ on the *x*-axis represents each patient’s journey through time from admission, with the time units being arbitrary and not representative of specific days. It should be noted that these curves are not absolute representations of mortality rates, but rather display the probability of survival over time for each group.

The predominant etiologies of septic shock varied significantly between groups: 40% of non-COVID-19 patients suffered from bacterial pneumonia, followed by intra-abdominal infections (30%). Conversely, the majority of COVID-19 patients developed septic shock secondary to viral pneumonia, often complicated by secondary bacterial infections. The mortality rates were notably different, with COVID-19 patients experiencing a higher mortality rate (42%), compared to non-COVID-19 patients (25%), reflecting the heightened severity observed in the former group elevated—Sequential Organ Failure Assessment (SOFA) scores: 9 vs. 6.

## 4. Discussion

The critical role of lactate in prognosticating outcomes in septic shock, as demonstrated in our study, resonates with the findings of Méndez et al. (2024), who underscored the prognostic significance of biomarkers like lactate in sepsis management across various clinical settings [2]. Our findings extend this understanding by elucidating the specific impact of COVID-19 on lactate dynamics, thereby enriching the existing literature on the biomarkers of sepsis. 

Our study revealed slight differences between the groups in terms of age. The COVID-19 patients group, associated with septic shock, presented a slightly lower average age. This observation may be related to the fact that the coronavirus disease was more prevalent in the elderly. Studies demonstrate that older people are more likely to develop severe acute respiratory syndrome coronavirus 2 (SARS-CoV-2) infection, and they have always been known to be at the highest risk of death from COVID-19. The severity and outcomes of COVID-19 largely depends on a patient’s age. Adults over 65 years of age represent 80% of hospitalizations and have a 23-fold greater risk of death than those under 65. From the clinical point of view, COVID-19 patients most commonly presented with fever, cough, and dyspnea, and from there, the disease can progress to acute respiratory distress syndrome, lung consolidation, cytokine release syndrome, endotheliitis, coagulopathy, multiple organ failure, and death [21]. At the same time, the characteristic symptoms of septic shock have several aspects in common with the symptoms of SARS-CoV-2 infection. Comorbidities such as cardiovascular disease, diabetes, and obesity increase the chances of fatal disease, but they alone do not explain why age is an independent risk factor. However, there are data which suggest that the age distribution of deaths in younger age groups (less than 65 years of age) was consistent across different settings and demonstrates how these data can provide robust estimates of the share of the population that has been infected. It was estimated that the infection fatality ratio was the lowest among 5–9-year-old children, with a log-linear increase by age among individuals older than 30 years [22].

A common pitfall was to rush to make a diagnosis when encountering a patient with COVID-19-like symptoms [23]. At the opposite pole, diagnosing septic shock without assessing the overlap of other potentially fatal diseases, such as SARS-CoV-2 infection, was risky during the defined time period in which this study was conducted. From the onset of the pandemic, various reports have indicated that, although there are some unique features pertinent to COVID-19, many of its acute manifestations are similar to sepsis caused by other pathogens [24]. This aspect influences patient management in the ED, as well the overall outcomes. Viral sepsis has some similarities but also some differences when compared to bacterial sepsis. In bacterial sepsis, systemic inflammation affecting multiple organs is more dominant than in COVID-19 sepsis. While bacterial sepsis causes an early and sudden onset clinical deterioration, viral diseases may exhibit a relatively late onset and chronic course. The consideration of severe COVID-19 disease as a sepsis syndrome has relevance and may assist in terms of determining the treatments that will modulate the immune response, limit intrinsic damage to tissue and organs, and potentially improve outcome.

In severe COVID-19 cases, laboratory parameters, such as hematological findings, coagulation tests, liver function tests, D-dimer, ferritin, and acute phase reactants such as CRP, present marked alterations, which are suggestive of a cytokine storm [25], changes that add and overlap with the sepsis-associated laboratory parameters modifications. The sepsis pathophysiological response involves different biochemical and immunochemical molecules that are expressed by different human tissues in molecular signaling pathways [26]. Laboratory parameters that present significant modifications in sepsis include lactate, CRP, cytokines, D-dimers, proadrenomedullin (ProADM). In addition to some of the classic biomarkers, such as CRP and PCT, several recent biomarkers which offer the potential to improve the diagnosis and treatment of sepsis were described: interleukin 6 (IL-6), soluble urokinase plasminogen activator receptor (suPAR), pro-adrenomedullin, presepsin, lipopolysaccharide binding protein, and a soluble triggering receptor expressed on myeloid cells (sTREM) [27]. However, considering the importance of lactate evaluation in sepsis, including the fact that the 2016 sepsis consensus definitions include lactate concentrations greater than 2 mmol/L (>18 mg/dL) as part of the definition of septic shock [28], our study focused on the evaluation lactate levels and additionally, on its clearance. The diagnostic and prognostic values of lactate in septic patients have been assessed in the setting of an emergency department, intensive care unit or in the trauma patient. High lactate is strongly associated with poor outcome and high mortality [29]. High lactate levels, associated with poor outcomes in our study, corroborate the existing literature on the subject and suggest that interventions aimed at improving lactate clearance could be beneficial in enhancing patient outcomes [30]. Emerging evidence on the predictive value of lactate clearance, not only in septic shock but also in other acute conditions, indicates the potential for lactate monitoring to play a broader role in critical care settings [31]. This highlights an opportunity for future research to explore innovative therapeutic interventions that enhance lactate clearance, such as optimizing fluid resuscitation techniques, employing early goal-directed therapy, or investigating new pharmacological agents that may influence lactate metabolism.

Elevated blood lactate levels in critically ill patients, including those with COVID-19, are traditionally considered as the indicators of impaired oxygen delivery to tissues. However, this simplification overlooks the complex interplay between tissue metabolic demands, oxygen supply, and clearance rates [30]. Lactate clearance and lactate levels have been established as prognostic markers in critically ill patients and patients following cardiac arrest, too [32,33]. In non-hypoxic conditions, elevated lactate can also result from increased aerobic glycolysis or the reduced activity of pyruvate dehydrogenase (PDH), highlighting the multifaceted nature of lactate production beyond mere oxygen insufficiency. This complexity underscores the importance of examining both lactate levels and the lactate-to-pyruvate (LP) ratio in critically ill COVID-19 patients, as these metrics offer augmented prognostic capabilities and insights into the underlying metabolic alterations. Studies suggest that an abnormal metabolic pattern, identified by elevated lactate levels or a high LP ratio, significantly correlates with higher ICU mortality rates among COVID-19 patients, emphasizing the role of metabolic dysfunction in patient outcomes. These findings suggest that the pathophysiological mechanisms driving lactate dynamics in COVID-19 may involve both oxygen delivery issues and metabolic factors like aerobic glycolysis and PDH activity. This distinction is crucial for understanding the unique metabolic stress experienced by COVID-19 patients in septic shock and may inform targeted therapeutic strategies to address these specific metabolic derangements [34].

Moreover, the COVID-19 pandemic has had broad implications for the management of septic shock, including challenges and opportunities for improving patient outcomes. Elevated blood lactate in septic patients, usually associated with mortality, does not necessarily relate to tissue hypoxia but may instead reflect mitochondrial dysfunction and high adrenergic stimulation. Interestingly, severe COVID-19 patients often exhibit near-normal blood lactate levels, suggesting preserved mitochondrial function despite a systemic hyperinflammatory state akin to sepsis. This nuanced understanding of the lactate dynamics in COVID-19 compared to other etiology sepsis cases underscores the need for adaptive management strategies that consider the unique pathophysiological mechanisms at play in COVID-19-induced sepsis [30]. Elevated lactate levels have traditionally been associated with hypoxia and poor outcomes, however, our study elucidates a nuanced role of lactate in septic shock, particularly influenced by COVID-19. This aligns with the findings from Vassiliou et al. (2022), which suggest that the pathophysiology of lactate elevation may extend beyond oxygen insufficiency to encompass metabolic alterations and mitochondrial dysfunction. These insights are pivotal as they propose that the lactate clearance mechanisms may vary significantly between COVID-19 and non-COVID-19 patients, emphasizing the need for tailored therapeutic strategies [30].

Our study highlights the prognostic value of lactate levels and clearance rates in septic shock patients, distinguishing between COVID-19 and non-COVID-19 cases. Similar to our observations, recent literature emphasizes the utility of lactate and lactate clearance as prognostic tools in septic shock management, defined by the Sepsis-3 criteria. Serum lactate levels, especially when measured at 6 h intervals, have been recognized as the effective predictors of patient outcomes, suggesting that both initial lactate levels and their subsequent clearance are critical in guiding treatment strategies and improving patient prognoses [30]. Moreover, studies have shown that lactate clearance, not just initial lactate levels, plays a crucial role in determining outcomes in septic shock patients. For instance, in a study focusing on patients with high bilirubin levels, those with increased lactate clearance showed significantly lower 28-day mortality, highlighting the importance of lactate clearance as a dynamic marker over time [35]. This aligns with our findings, underscoring the need for close monitoring of lactate clearance rates as part of comprehensive patient management in the emergency department.

The emerging evidence on lactate dynamics in septic shock, particularly in the context of COVID-19, calls for a multi-faceted exploration into future research and interventions. Shankar-Hari et al. (2016) highlight the nuanced understanding of sepsis definitions and clinical criteria, suggesting the need for continued refinement in diagnostic and treatment strategies to address septic shock more effectively [36]. Furthermore, Allo et al. (2023) demonstrate the potential of lactate clearance as a predictive tool in acute conditions like upper gastrointestinal bleeding, indicating the broader applicability of lactate metrics in critical care settings [37]. These insights pave the way for innovative therapeutic interventions aimed at enhancing lactate clearance, potentially improving outcomes in septic shock patients. By comparing the lactate levels between COVID-19 and non-COVID-19 patients, our study provides a unique lens on the metabolic complexities introduced by the pandemic. The differential impact on lactate clearance rates not only supports but also expands on studies like those by Lee et al. (2021), which demonstrate that COVID-19 complicates the sepsis landscape, thus necessitating a re-evaluation of the existing management protocols [3].

While our study contributes valuable insights into the prognostic significance of lactate levels and clearance in septic shock, it is not devoid of limitations. The reliance on observational data from a single center may limit the generalizability of our findings across diverse clinical settings and populations. Future studies should aim to elucidate these mechanisms and explore the impact of different therapeutic interventions on lactate clearance and patient outcomes. Other study limitations, like the random selection process of the patients and the lack of consideration of other risk factors, should be noted.

This study highlights the critical role of lactate levels and lactate clearance in predicting outcomes for patients with septic shock, with an emphasis on the nuances introduced by COVID-19. The novelty of this study lies in the detailed comparison between patients with COVID-19 septic shock and those not affected by COVID-19, demonstrating that dairy dynamics have distinct prognostic implications in the context of the pandemic. Our findings reinforce the importance of early and continuous lactate monitoring in the management of septic shock, emphasizing the need for aggressive interventions aimed at improving lactate clearance to improve patient outcomes.

## 5. Conclusions

This study underscores the significant role of lactate levels and clearance as prognostic indicators in septic shock, with a specific emphasis on the differences observed between COVID-19 and non-COVID-19 patients. Through comparative analysis, it reveals that lactate dynamics have unique implications for predicting patient outcomes in the context of the pandemic. By advocating for early and diligent lactate monitoring, our research contributes to a deeper understanding of septic shock prognostics, encouraging further explorations into how these insights can be applied across diverse patient populations.

## Figures and Tables

**Table 1:** Demographics data. *p*-value < 0.05 significant, Student *t*-test.

Variable	Group 1 Non-COVID-19 *n* = 23, *n* (%)	Group 2 COVID-19 *n* = 91, *n* (%)	*p*-Value
Age	67 ± 18.41	71 ± 15.85	0.031
Gender (male)	17 (74%)	67 (74%)	0.712
Gender (female)	6 (26%)	24 (26%)	0.524
Comorbidity and complications			
Heart failure, *n* = 10	1 (4%)	9 (9%)	0.045
COPD, *n* = 31	13 (56%)	18 (19%)	0.241
CKD, *n* = 6	2 (8%)	4 (4%)	0.642
Diabetes, *n* = 17	8 (34%)	9 (9%)	0.264
Cardiovascular diseases (CVDs), *n* = 48	21 (91%)	27 (29%)	0.021
Oncological disease, *n* = 7	3 (30%)	4 (4%)	0.854
Hematologic disease, *n* = 3	1 (4%)	2 (2%)	0.457
Neurological disease, *n* = 3	1 (4%)	2 (2%)	0.243
Obesity, *n*= 24	7 (30%)	17 (18%)	0.031
Multi-organ failure, *n* = 11	1 (4%)	10 (10%)	0.001

**Table 2:** Laboratory Parameters. CRP: C-reactive protein; WBC: white blood count; PCT: Procalcitonin. Normal level: lactate 0.5–2.1 mmol/L; CRP < 0.5 mg/dL; PCT < 0.5 ng/mL.

Laboratory Parameters	Group 1 (Non-COVID-19)	Group 2 (COVID-19)	*p*-Value
Lactate start, mmol/L	4.7 ± 2.6	6.2 ± 2.9	0.001
Lactate 6 h, mmol/L	4.5 ± 2.3	5.7 ± 2.4	0.021
Lactate 24 h, mmol/L	3.7 ± 1.9	4.3 ± 2.1	0.037
Lactate 48 h, mmol/L	2.3 ± 1	3.2 ± 1.4	0.046
Lactate clearance at 6 h (%)	29 ± 23	27 ± 24	0.054
Lactate clearance at 24 h (%)	35 ± 17	33 ± 12	0.017
WBC, mm^3^	14.7 ± 4.2	15 ± 4.6	0.078
CRP mg/dL	134.4 ± 18.9	189.2 ± 24.4	0.064
PCT ng/mL	9.7 ± 4.1	17.2 ± 3.7	0.049
pH	7.29 ± 0.24	7.31 ± 0.14	0.241
pO_2_, mmHg	64.4 ± 21.5	60.9 ± 15.3	0.045
pCO_2_, mmHg	50.4 ± 19.6	53.4 ± 21.6	0.069

**Table 3:** Comparison of the lactate clearance between survivors and non-survivors after 72 h in septic shock patients.

Patient Group	Survivors after 72 h, *n* = 81	Non-Survivors after 72 h, *n* = 33	*p*-Value
Lactate mmol/L	1.5 ± 0.4	2.5 ± 0.5	0.001
Lactate clearance (%)	33 ± 15	24 ± 9	0.002

## Data Availability

The raw data supporting the conclusions of this article will be made available by the authors on request.
